# Treatment of Tuberculous Meningitis With Contezolid in a Patient With Complex Comorbidities: A Case Report and Literature Review

**DOI:** 10.1155/crdi/8814569

**Published:** 2025-08-09

**Authors:** Qi Fan, Hong Sun, Shengsheng Liu

**Affiliations:** Department of Tuberculosis, Anhui Chest Hospital, Hefei 230022, Anhui, China

**Keywords:** chronic kidney failure, contezolid, *Mycobacterium tuberculosis*, pancytopenia, tuberculous meningitis

## Abstract

Central nervous system (CNS) tuberculosis (TB) is a severe manifestation of *Mycobacterium tuberculosis* (MTB) infection, characterized by high mortality. Contezolid, a novel oxazolidinone antibiotic, exhibits in vitro activity against MTB and may offer a safety advantage over linezolid, a first-generation oxazolidinone frequently linked to myelosuppression and neuropathy. Clinical data on contezolid in CNS tuberculosis remain scarce. We report a middle-aged man with chronic renal allograft dysfunction who was receiving long-term hemodialysis and subsequently developed severe CNS TB complicated by multiple coinfections, diabetes mellitus, and pancytopenia. An individualized multidrug regimen that included contezolid was successfully employed, suggesting its potential utility in complex CNS TB. This report highlights therapeutic considerations for similar patients and underscores the need for further research on the role of contezolid in TB treatment.

## 1. Introduction

Central nervous system (CNS) tuberculosis (TB) represents the most severe form of extrapulmonary disease, often resulting in irreversible neurological sequelae such as encephalitis, cranial neuritis, hydrocephalus, vasculitis, and stroke, and is associated with substantial mortality [1]. Although current treatment combines antituberculosis (anti-TB) agents with anti-inflammatory therapy, optimal efficacy remains elusive, prompting interest in novel antibiotics or improved drug delivery to the site of infection. Agents with high cerebrospinal fluid (CSF) penetration, such as linezolid, have demonstrated efficacy in CNS TB and are recommended for multidrug-resistant (MDR) and extensively drug-resistant (XDR) TB [2]. However, linezolid's use is limited by adverse effects, including peripheral and optic neuropathy and bone-marrow suppression [3]. Contezolid, a next-generation oxazolidinone, was originally developed for MDR Gram-positive infections, including complicated skin and soft-tissue infections caused by *Staphylococcus aureus*, Streptococcus pyogenes, and Streptococcus agalactiae [4–6]. In vitro susceptibility testing has shown that contezolid possesses activity comparable to linezolid against both drug-susceptible and drug-resistant *Mycobacterium tuberculosis* (MTB) [7, 8], while emerging clinical data indicate a favorable safety profile with lower rates of myelosuppression and neurotoxicity [9–11]. Notably, contezolid does not require dose adjustment in mild-to-moderate renal impairment [12, 13], and its tolerability has been confirmed in patients receiving hemodialysis during short antibacterial courses [14]. A previous report documented successful treatment of recurrent refractory TB ascites with contezolid in a patient with chronic kidney transplant dysfunction [15]. Despite these promising findings, the clinical applicability and safety of contezolid, particularly concerning its distinct metabolic properties, require further validation. Here, we describe a middle-aged man with severe CNS TB and chronic renal allograft dysfunction on long-term hemodialysis, complicated by diabetes, pancytopenia, and polymicrobial infections, who responded favorably to an individualized multidrug regimen including contezolid. This report aims to broaden the understanding of anti-TB strategies in patients with complex comorbidities.

## 2. Case Presentation

**Figure 1:**
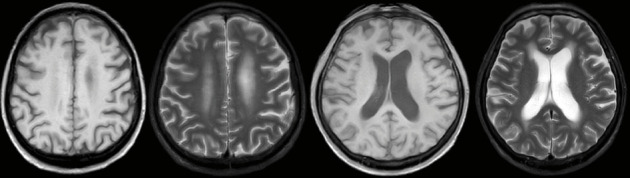
Brain magnetic resonance imaging (MRI) scan upon admission.

A 62 year-old man with a 16 year history of well-controlled Type 2 diabetes mellitus, a 10 year history of hypertension, and a 13 year history of chronic kidney disease (on thrice-weekly hemodialysis for the past 4 years) was admitted on 20 October 2023 with a 4 day history of altered consciousness and an 11 day history of intermittent fever that had initially been overlooked until neurological deterioration prompted prior hospitalization, where empirical antimicrobial therapy proved ineffective. Three years after total gastrectomy for gastric cancer, he was transferred to our intensive care unit (ICU) with pancytopenia (white blood cells [WBCs]: 1.90 × 10^9^/L, red blood cells [RBCs]: 4.11 × 10^12^/L, and platelets [PLTs]): 9 × 10^9^/L), hypoalbuminemia (31.7 g/L), and acute-on-chronic renal failure (creatinine: 445.9 μmol/L). Brain magnetic resonance imaging (MRI) revealed multifocal abnormal signals in the bilateral frontoparietal cortices and semioval centers (Figure 1), while chest computed tomography (CT) showed miliary nodules, left upper-lobe consolidation, and right lower-lobe bronchiectasis. CSF analyzed by metagenomic next-generation sequencing (mNGS) yielded *MTB* DNA, confirming tuberculous meningitis. Bronchoalveolar lavage fluid (BALF) obtained via bedside bronchoscopy was positive on GeneXpert MTB/RIF (rifampicin-susceptible, “low” detection level); mNGS additionally identified *Streptococcus pneumoniae, Pseudomonas aeruginosa, Aspergillus, Pneumocystis jirovecii*, and *MTB* DNA sequences, supporting miliary TB. Subsequent cultures of both BALF and CSF confirmed growth of *MTB*, while BALF also yielded *Aspergillus fumigatus* with a positive galactomannan (GM) antigen test, indicating concomitant invasive pulmonary aspergillosis. Final diagnoses were CNS TB, miliary pulmonary tuberculosis (PTB), invasive pulmonary aspergillosis, and polymicrobial pulmonary coinfection.

Owing to preexisting bone-marrow suppression, linezolid, known to worsen cytopenias, was avoided in favor of contezolid. Because voriconazole was required for concurrent fungal infections and rifampicin markedly reduces voriconazole plasma concentrations [16], rifampicin was excluded from the initial regimen. The anti-TB regimen comprised isoniazid 300 mg once daily, pyrazinamide 1500 mg once daily, contezolid 400 mg twice daily, and cycloserine 250 mg twice daily, supplemented with voriconazole 200 mg twice daily for antifungal therapy. All medications were administered after hemodialysis sessions. PLT transfusions and other hematopoietic supportive measures were provided. The patient improved clinically within 6 days, with resolution of fever and restoration of consciousness. Follow-up laboratory tests showed hematological recovery: WBCs: 5.09 × 10^9^/L, RBCs: 4.13 × 10^12^/L, and PLTs: 54 × 10^9^/L. After stabilization, he was discharged to continue thrice-weekly hemodialysis.

**Figure 2:**
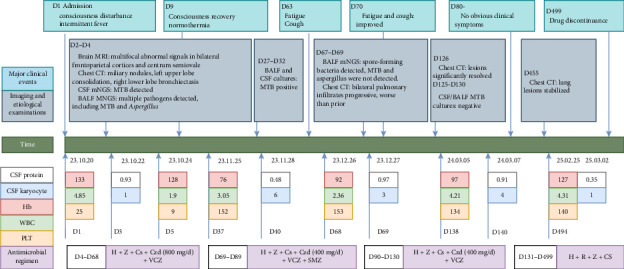
Overview of key clinical events, examination results, and treatment regimens. Abbreviations: BALF: bronchoalveolar lavage fluid; Cs: cycloserine; CSF: cerebrospinal fluid; Czd: contezolid; D: patient day(s); H: isoniazid; Hb: hemoglobin; mNGS: metagenomic next-generation sequencing; MRI: magnetic resonance imaging; MTB: *Mycobacterium tuberculosis*; PLT: platelets; R: rifampicin; RBC: red blood cells; SMZ: sulfamethoxazole-trimethoprim; WBC: white blood cells; VCZ: voriconazole; Z: pyrazinamide.

**Figure 3:**
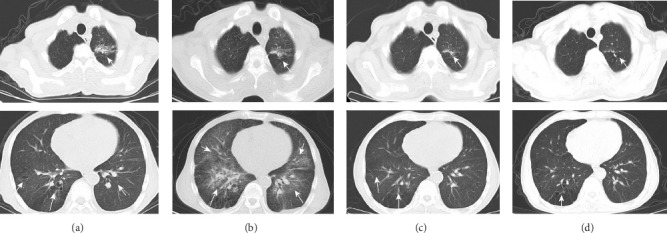
Serial chest computed tomography (CT) findings throughout the treatment course. (a) Chest CT image upon admission. (b) Chest CT showing progression of infection. (c) Chest CT demonstrating improvement following treatment. (d) Final CT at treatment completion revealing stable fibrotic changes without active disease. White arrows indicate lesions.

At the 2-month follow-up, the patient reported fatigue and a new-onset cough of 1-week duration, without fever or neurological symptoms. Examination revealed no recurrence of CNS infection. CSF parameters remained stable compared to previous results (Figure 2), and cultures were negative, confirming controlled tuberculous meningitis. Chest CT showed progressive bilateral pulmonary infiltrates (Figure 3(b)). BALF mNGS detected spore-forming bacteria, prompting initiation of cotrimoxazole 960 mg three times daily after respiratory consultation. Drawing on the experience of linezolid dose adjustment in TB treatment in the literature [17] and considering the patient's limited financial resources, we reduced contezolid to 400 mg once daily. After 3 weeks of therapy and clinical improvement, cotrimoxazole was discontinued. Subsequent chest CT on 22 February 2024 demonstrated marked lesion resolution (Figure 3(c)). At the March review, he remained asymptomatic with normal findings. CSF profiles were stable from baseline (Figure 2), with negative MTB cultures from CSF and BALF, and no pathogens detected by mNGS. After clinical assessment confirmed cure of pulmonary aspergillosis, voriconazole was stopped, and anti-TB therapy was adjusted to rifampicin 600 mg once daily, isoniazid 300 mg once daily, pyrazinamide 1500 mg once daily, and cycloserine 250 mg twice daily. Follow-up has shown continued clinical stability, with chest CT on 17 January 2025 revealing unchanged pulmonary findings (Figure 3(d)). Throughout treatment, WBC and PLT counts fluctuated within safe ranges, while CSF cellularity and protein normalized (Figure 2). All microbiological results are summarized in Table 1. Key clinical events, examination findings, and treatment regimens are depicted in Figure 2. Anti-TB therapy was completed on 2 March 2025, with ongoing surveillance planned.

## 3. Discussion

This complex case involved concurrent CNS and PTB with polymicrobial coinfections, further complicated by diabetes mellitus, hypertension, and end-stage renal disease requiring maintenance hemodialysis. Unexplained pancytopenia posed additional therapeutic challenges, necessitating deviation from standard anti-TB protocols. The patient's multifaceted pathology demanded an individualized strategy that (1) tailored antimicrobial selection to address both tuberculosis and opportunistic infections while accommodating renal impairment, (2) carefully managed drug interactions, particularly between anti-TB and antifungal agents, and (3) ensured ongoing hematological monitoring given baseline cytopenias. This approach balanced microbiological efficacy with organ-system preservation, underscoring the need for personalized management in complex tuberculosis presentations.

Designing the initial anti-TB regimen was challenging due to the patient's complex clinical status, requiring individualization over standardization. Rifampicin was excluded initially due to its significant interaction with voriconazole [16]. CSF penetration guided agent selection; the initial regimen included isoniazid, pyrazinamide, and cycloserine, with an oxazolidinone added later. While linezolid offers strong CSF penetration and anti-TB activity, its risk of myelosuppression was problematic, given the patient's pancytopenia. We thus chose contezolid, a domestically developed oxazolidinone approved in 2021 for complex skin and soft-tissue infections [18], for its favorable safety profile. Contezolid's distinctive chemical structure, featuring a nonplanar A- and B-ring system conferred by an orthofluoro substituent, is associated with reduced myelosuppressive toxicity [19]. Clinical evidence demonstrates resolution of linezolid-associated peripheral neuropathy and hematological toxicity after switching to contezolid [9, 15, 20], while in vitro studies by Wang et al. revealed superior intracellular bactericidal activity against MTB compared to linezolid [21]. After a comprehensive discussion and informed consent from the patient's family, contezolid was incorporated into the anti-TB regimen.

To our knowledge, published reports of contezolid in CNS tuberculosis remain extremely limited. A literature search of PubMed from 1 January 2015 to 31 January 2025 using the terms “contezolid” and “tuberculosis” identified only three documented cases, including ours (Table 2; Supporting Figure 1). Among these, our patient presented the most complex clinical picture and posed the greatest therapeutic challenge. The case series comprised one male and two female patients (mean age: 54.3 years, range: 32–69). One case shared similar neurological manifestations, fever and altered consciousness [22], with our patient, whereas another exhibited only constitutional symptoms such as fever, nausea, and vomiting [20]. Notably, our case is the first in which contezolid was used as initial anti-TB therapy, whereas the other two involved substitution after linezolid intolerance. A further distinguishing feature is that our patient had pancytopenia while receiving long-term hemodialysis. The cytopenia most likely resulted from the combined effects of severe infection, renal anemia, and uremic thrombocytopenia rather than drug toxicity. After initiation of an anti-TB regimen containing contezolid 400 mg twice daily, no additional myelosuppression occurred; instead, blood counts gradually improved alongside infection control and adjunctive hematopoietic support. Although blood drug concentrations were not monitored, therapeutic efficacy was not compromised by hemodialysis. This observation may provide valuable guidance for treating TB in patients with renal failure requiring hemodialysis. The maximum reported duration of contezolid therapy was 8 months [22], with no drug-related adverse events in any case, suggesting its potential as a safer alternative to linezolid. Although anti-TB regimens differed, all three patients achieved favorable outcomes, indicating that contezolid is both effective and well tolerated in CNS TB. Pharmacokinetic data remain sparse, with only one case reporting CSF concentrations: at 7 h postdose during Weeks 7 and 11, serum levels were 9.64 mg/L and 9.36 mg/L, respectively, and corresponding CSF concentrations were 0.54 mg/L and 1.15 mg/L [20]. The limited sampling underscores the need for more comprehensive pharmacokinetic studies assessing tissue penetration, as well as randomized controlled trials to establish contezolid's role in CNS TB. Interestingly, rifampicin was omitted during a specific stage in all three cases for various reasons, yet successful outcomes were achieved, further supporting the potential inclusion of contezolid in CNS TB regimens.

Our investigation has two principal limitations. First, the absence of CSF and blood concentration monitoring for contezolid precludes definitive dose optimization for CNS TB. We plan to collect paired serum and CSF samples on Day 8 and at Weeks 4, 6, and 8 of therapy for pharmacokinetic analysis. Second, the patient's chronic hemodialysis complicated the accurate assessment of contezolid's nephrotoxic potential. Currently available pharmacokinetic data are insufficient to establish optimal dosing and administration timing for patients with renal impairment requiring dialysis, an important knowledge gap that warrants further study.

Despite these limitations, our findings suggest that contezolid may be a potentially valuable therapeutic alternative for CNS TB patients, who require hemodialysis and have baseline pancytopenia. These preliminary observations are hypothesis-generating; adequately powered, controlled studies involving larger cohorts are needed to confirm contezolid's role in TB management, particularly among patients with renal impairment or hematological vulnerability.

## Figures and Tables

**Table 1:** The results of the patient's etiological examination during the course of the disease.

Etiology	Before treatment			After 2 months of treatment			After 4.5 months of treatment			After 15 months of treatment		
	Sputum	BALF	CSF	Sputum	BALF	CSF	Sputum	BALF	CSF	Sputum	BALF	CSF
*Mycobacterium tuberculosis*	+	+	+	−	−	−	−	−	−	−	N/A	−
*Aspergillus*	−	+	−	−	−	−	−	−	−	−	N/A	−
*Streptococcus pneumoniae*	−	+	−	−	−	−	−	−	−	−	N/A	−
*Pseudomonas aeruginosa*	−	+	−	−	−	−	−	−	−	−	N/A	−
*Pneumocystis jirovecii*	−	+	−	−	−	−	−	−	−	−	N/A	−
*Pneumocystis jiroveci*	−	−	−	−	+	−	−	−	−	−	N/A	−

*Note:* BALF, bronchoalveolar lavage fluid; CSF, cerebrospinal fluid.

Abbreviation: N/A = not applicable.

**Table 2:** Summary of reported features of contezolid in the treatment of central nervous system tuberculosis cases.

Source	PMID	Age (years)	Sex	Symptoms at admission	Comorbidities	Antituberculosis regimen	Timing of contezolid use	Monitoring of contezolid concentration in CSF	Duration of contezolid use	Contezolid-related adverse reactions	Other important treatment	Outcome
Our case	/	62	Male	Fever and disturbances in consciousness	Chronic kidney failure, diabetes mellitus, hypertension, gastric cancer, and pancytopenia	HZCsCzd, HRZCs	Initial use	No	4.5 months	None	Antifungal treatment, treatment of pancytopenia, and hemodialysis	Improved
Guo et al. [20]	37276894	32	Female	Fever, headache, nausea, and vomiting	None	HRZEMfx, HRZMfxLzd, HZBdqMfxLzdFPM, HZBdqMfxCzdFPM	Replacement for linezolid	Yes	More than 11 weeks, but the specific time is unknown	None	Unknown	Improved
Xu et al. [22]	37928457	69	Female	Fever, slurred speech, ptosis of the left eyelid, and loss of consciousness	Acute renal failure, liver injury, and anemia	HRZELzd, HRELfxLzd, HELfxLzdCs, HELfxCzdCs, HLfxCzd	Replacement for linezolid	No	8 months	None	Liver-protecting treatment, transfusion, and hemodialysis	Improved

*Note:* Bdq, bedaquiline; Cs, cycloserine; Czd, contezolid; E, ethambutol; FPM, faropenem; H, isoniazid; Lfx, levofloxacin; Lzd, linezolid; Mfx, moxifloxacin; R, rifampin; Z, pyrazinamide.

## Data Availability

The data that support the findings of this study are available from the corresponding author upon reasonable request.

## Supporting Information

The retrieval process for literature review: A PubMed search using the keywords “contezolid” and “tuberculosis” yielded 24 articles. After screening titles and abstracts, 2 articles were excluded due to irrelevance. Full-text reviews further excluded 19 articles not involving CNS infections and 1 article on NTM infection, resulting in 2 target articles. Supporting Figure 1: The retrieval process for literature review. Abbreviations: CNS: central nervous system; NTM: non-*tuberculous Mycobacteria*.
