# Supplementary figures and images for: Treatment of Tuberculous Meningitis With Contezolid in a Patient With Complex Comorbidities: A Case Report and Literature Review
Source: Case Rep Infect Dis. 2025 Aug 9;2025:8814569. doi: 10.1155/crdi/8814569 (PMC12357779; doi:10.1155/crdi/8814569)

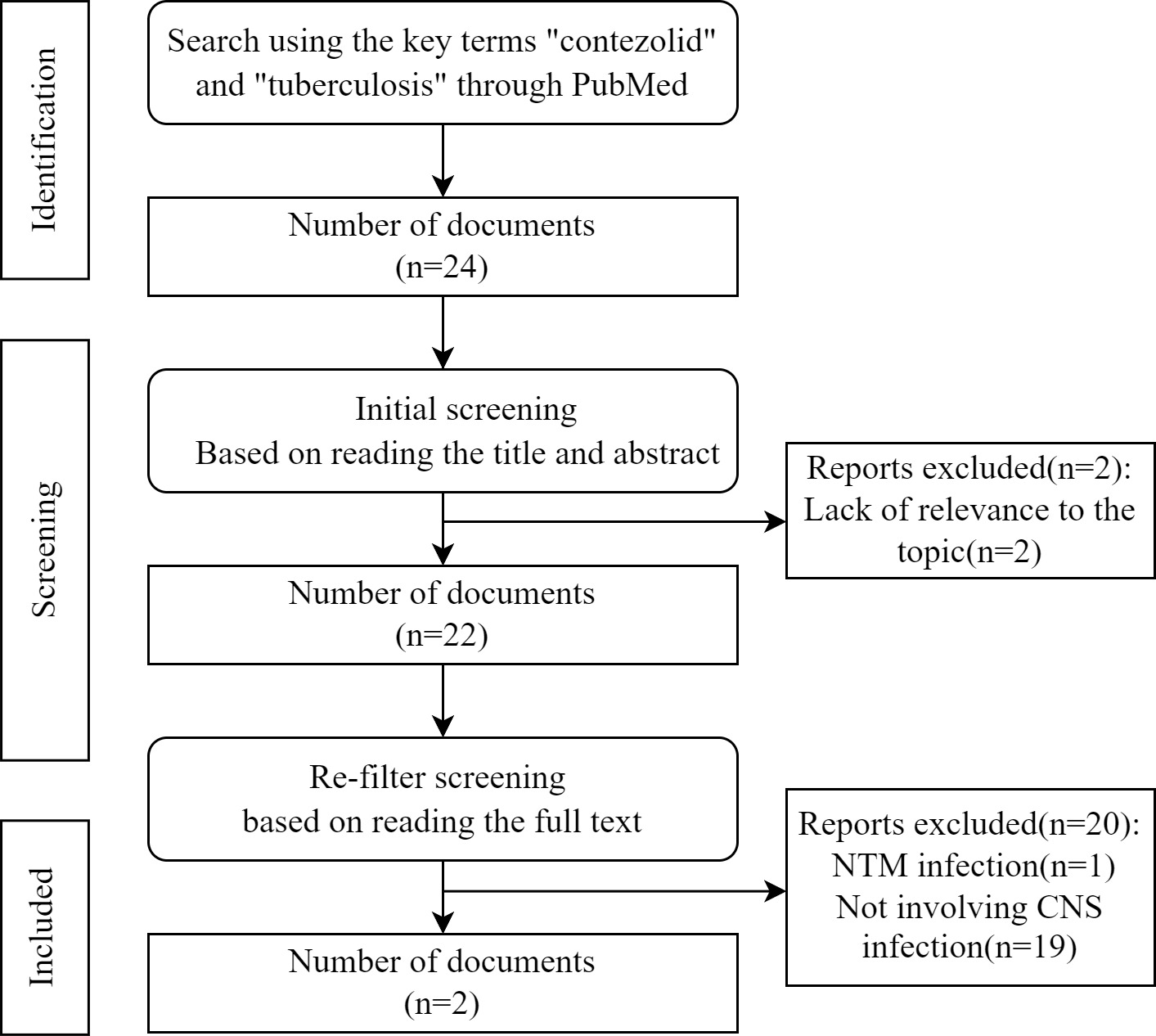

Supplement: Supporting Information — Additional supporting information can be found online in the Supporting Information section. [file 8814569.f1.jpg]
